# Intake of Vitamins and Minerals From Voluntarily Fortified Foods and/or Dietary Supplements in School Adolescents in Central-Eastern Poland

**DOI:** 10.3389/fpubh.2020.504015

**Published:** 2020-10-09

**Authors:** Ewa Sicińska, Barbara Pietruszka, Olga Januszko, Sebastian Jakubowski, Kamila Kielak-Biskupska, Katarzyna Rolf, Joanna Kaluza

**Affiliations:** Department of Human Nutrition, Institute of Human Nutrition Sciences, Warsaw University of Life Sciences (WULS—SGGW), Warsaw, Poland

**Keywords:** adolescents, dietary supplements, fortified foods, minerals, vitamins

## Abstract

**Background:** The key issue is whether voluntarily fortified foods and vitamin/mineral supplements available on the market serve public health needs. The study aim was to estimate nutrient intakes from voluntarily fortified foods and vitamin/mineral supplements in relation to the Dietary Reference Intake (DRI) in adolescents (*n* = 759) aged 13–19 who attended public secondary schools in Central-Eastern Poland.

**Methods:** Data on the consumption of voluntarily fortified foods were collected using a semi-quantitative food frequency questionnaire containing 58 food items. Data on the use of dietary supplements were assessed via an open-ended question. The content of nutrients was estimated using the producer's labeling declaration. The distribution of nutrient intakes according to the percentage of DRI categories (<20%, 20–39.9%, 40–59.9%, 60–79.9%, 80–99.9%, 100–119%, or >120%) was estimated.

**Results:** Consumption of voluntarily fortified foods was a common behavior in adolescents (86.7% of participants), while vitamin/mineral supplements were used by less than one-fifth of them (17.7%). The amounts of nutrient intakes from fortified foods and/or supplements were at different levels: (I) vitamins A, D, calcium, magnesium (>50% of adolescents did not exceed 20% of DRI); (II) vitamins E, B_12_, iron (>50% of respondents consumed at least 20% of DRI); (III) niacin and pantothenic acid (>50% of respondents consumed at least 40% of DRI); IV) vitamins C, B_1_, B_2_, B_6_, folate, biotin (>50% of participants consumed at least 60% of DRI). In a subgroup of respondents who used fortified foods and supplements simultaneously (*n* = 126), some nutrients (i.e., vitamins C, B_1_, B_2_, B_6_, niacin, and biotin) were consumed in amounts ≥150% of DRI. Intake above the Tolerable Upper Intake Levels was observed for niacin, vitamin A, B_6_ and folic acid in individual cases (up to 1.1% of respondents); a higher risk of overconsumption was associated with using vitamin/mineral supplements than voluntarily fortified foods.

**Conclusion:** Adolescents should be educated on how to reasonably use fortified foods and dietary supplements to help to overcome the potential deficiency of nutrients without causing excessive consumption.

## Introduction

The intake of most vitamins and minerals in European countries is generally adequate; however, the risk of a low intake of some nutrients (e.g., vitamin D, iron, and iodine) is likely to appear in specific population subgroups, such as children and adolescents (1). Food fortification and dietary supplementation are effective strategies for tackling nutritional deficiencies; however, a reasonable approach to using these products is to support, but not replace, a well-balanced diet.

Food fortification (any addition of nutrients to food in the manufacturing process) is a solution to address micronutrient deficiencies at a population level and simultaneously does not require changes in the diet. Mandatory fortification has been practiced over several decades in Western countries as well as in the developing world to target specific health conditions, e.g., iodised salt to avoid goiter; B-vitamins and iron-enriched cereals for people with anemia diagnosis; folic acid-fortified cereals to reduce the risk of pregnancy affected by neural tube defects. On the other hand, voluntary food fortification (at the discretion of food manufacturers) depends on how well producers know and respond to the nutritional needs of vulnerable population groups. A market-driven fortification can play a positive role in public health nutrition, but it could be focused on the marketing advantage of the company as well; therefore, it may not meet the requirements of the nutritional policy (2). In general, children and adolescents consume fortified foods more often than adults (3, 4); this is reflected by the substantial amount of foods designed for younger populations that are available on the market, such as vitamin-fortified candies or carbonated beverages, which are not recommended by dieticians (4–6). A large number of voluntarily fortified foods (VFFs) on the market makes it necessary to choose products that could effectively complement nutritional deficiencies, which demands nutritional knowledge from consumers. An increased VFF consumption can also be associated with a greater risk of high nutrient exposure in some population subgroups (7). Therefore, the essential question is whether the VFFs that are present on the market serve public health needs in industrialized countries.

Food supplements (concentrated sources of nutrients in a single dose form) have the potential to quickly deal with specific nutritional deficiencies at an individual level; however, there are often concerns about the relatively high micronutrient content of vitamin/mineral supplements (VMSs), which can increase the risk of deliberate or accidental over-consumption (2).

Despite the uniform European regulations (8, 9), fortification and supplementation practices differ greatly between European countries. The market of VFFs and VMSs is developing dynamically; therefore, information on consumption in various European regions may be underestimated. In the national representative dietary surveys conducted in eight European countries (including Poland), information on VFF consumption was very limited, and was accurately assessed only in Germany and the Netherlands as well as partially for particular foods in three others countries. Data on the prevalence of VMSs seems to be more accurate, but there were differences in defining VMS users as well as the research methodology and the frequency of using these products in different age groups across Europe (1).

To help tailor strategies for meeting nutrient recommendations, it is very important to evaluate the consumption of nutrients from VFFs and/or VMSs in young people at whom these products are often targeted. Therefore, the project on the use of VFF and VMS among school children and adolescents was carried out in Central-Eastern Poland. Our previous publications identified socio-demographic and lifestyle determinants, influencing the use of these products in a group of children and adolescents (*n* = 1,578, aged 5–20 years) (10, 11), and examined nutrient intakes from VFF in a subgroup of the children (*n* = 677, aged 6–12) (11). As a part of this project, we estimated vitamin and mineral intakes from additional sources (VFF and/or VMS) in relation to the Dietary Reference Intake (DRI) of a subgroup of adolescents. The study proposes a new approach to the classification of nutrient intake from additional sources in the context of complementation. Moreover, we analyzed whether there is a potential risk of nutrient overdose from these products.

## Materials and Methods

### Study Design

The cross-sectional study was conducted among 13–19-year-old adolescents who attended schools in Central-Eastern Poland. The schools were selected randomly and located in four provinces: Mazowieckie, Wielkopolskie, Łódzkie, and Kujawsko-Pomorskie. The largest number (40%) of respondents lived in Mazowieckie, which has the greatest adolescent population of all provinces (12). Twenty-five public secondary schools were invited to participate in the study; however, the survey was conducted only in those schools where the headteacher gave their consent. The research was conducted in cooperation with the schools' teachers. The inclusion criteria of participating in the study were: attendance to a public lower or higher secondary school in the Mazowieckie, Wielkopolskie, Łódzkie, or Kujawsko-Pomorskie provinces; and the age of respondents from 13 to 19 years old. The criteria of exclusion were: the presence of a disease requiring special dietary treatment (e.g., celiac disease, diabetes, chronic kidney, and liver diseases); pregnancy or lactation; and incomplete or incorrectly filled out questionnaires (e.g., difficulty with product identification, lack of portion sizes of consumed products, the frequency of consumption of product multiply marked per month and week).

All procedures performed in the study were in accordance with the 1964 Helsinki declaration and its later amendments. According to Polish legislation, the approval of an ethical committee was not required for non-invasive studies before 2016. In 2016, the study protocol was registered and approved by the Ethical Committee of the Warsaw University of Life Sciences (Resolution No. 09_1/2016). Parents were informed about the children's participation in the conducted survey. By completing the questionnaires, the respondents agreed to participate in the study.

### Study Population and Data Collection

The data was collected using a self-administered health and lifestyle questionnaire and a semi-quantitative food frequency questionnaire (FFQ) of voluntarily fortified products with vitamins and/or minerals. The questionnaires were distributed among 1,200 adolescents during classroom sessions. The respondents were instructed on how to complete the questionnaires and they were asked to return them to the schools' teachers within 1 week. A total of 846 respondents returned the questionnaires (response rate of 70.5%); however, 36 participants did not meet the age inclusion criteria, and 51 participants were excluded due to an incorrectly completed health and lifestyle questionnaire and/or FFQ. Finally, 759 adolescent were included in the study.

The health and lifestyle questionnaire included 27 questions contained in the following sections: socio-demographic characteristics, health and lifestyle status, eating habits, VMS and VFF usage. The participants were asked whether they used VMSs during the year before the study. Information on the name and the brand of dietary supplements, the form of preparation (capsules, tablets, powder etc.), the period of application and daily doses, were collected. All subjects who reported that they used at least one VMS within at least 1 week or longer over the past 12 months were classified as VMS-users. Moreover, the respondents were asked to point out whether they consumed VFFs over the past month; the definition of these products was provided in the health and lifestyle questionnaire to help the participants understand the questions. Details of the study methods have been provided elsewhere (10). Briefly, the health and lifestyle questionnaire was based on a previously developed questionnaire used in a study among 1,019 adult respondents from Central and Eastern Poland (13), and was adapted for different age groups, including children (14), adolescents and students (15–17).

The FFQ was used to verify the information given in the health and lifestyle questionnaire about VFF consumption as well as to assess the accurateness of the collected data. The FFQ contained 58 food items (brands available on the Polish market) divided into the following 6 categories: cereal products (16 items), dairy products (six items), juices and non-alcoholic beverages (15 items), instant beverages (10 items), sweets (nine items), and desserts (two items). The mandatory fortified foodstuffs, i.e., iodised table salt and fat spreads with added vitamin A and D (18), were not included in the FFQ list. In the case that, during the study, some new products appeared on the market, the respondents could add extra products to each category of VFF products. In order to facilitate respondents completing the questionnaire, an example of how to fill out the questionnaire was provided. The participants indicated how many times daily/weekly/monthly they usually consume specific food items and indicated the average portion size (in grams/milliliters or household measures). All subjects who reported the consumption of at least one of the products from the FFQ list were classified as VFF-consumers. The FFQ questionnaire was validated using 5-day dietary records among 102 students (19). The Bland-Altman agreement analysis between the two methods used indicated good limits of agreement (LOA) for all groups of VFF products (except desserts); the LOAs were from 1.96% for sweets to 4.90% for cereal products (and 5.88% for desserts). The Spearman correlation coefficients between estimates from the FFQ and the diet records were from 0.32 for desserts to 0.58 for dairy products.

### Nutrient Intakes From VFFs and/or VMSs

Based on the collected data, the numbers of adolescents that consumed certain nutrients from additional sources (i.e., VFF and/or VMS) were assessed. The nutrients consumed by more than 5% of participants (i.e., vitamin A, vitamin E, vitamin D, vitamin C, vitamin B_1_, vitamin B_2_, niacin, vitamin B_6_, folic acid, vitamin B_12_, pantothenic acid, calcium, magnesium, and iron) were included in the analysis. The amounts of vitamin and mineral intakes from VFFs and/or VMSs were calculated based on the frequency and quantity of their consumption and the content of the nutrients listed on the producer's labeling declarations. The amounts of nutrients consumed from VFFs and/or VMSs were compared to the Polish DRI, i.e., Estimated Average Requirement (EAR) or Adequate Intake (AI), individually for each respondent depending on age and sex (20). Due to the fact that data on usual diet consumption was not collected, vitamin and mineral intakes from additional sources were presented in relation to the percentage of the DRI instead of using the probability approach to estimate the prevalence of nutrient inadequate intake. Moreover, to determine whether the risk of consuming excessive amounts of nutrients has existed, the data were compared to the Tolerable Upper Intake Levels (UL) values individually for each participant; for vitamins A, E, D, B_6_ and folic acid, the values established by the European Food Safety Authority (21, 22), and for vitamin C, niacin, calcium, magnesium and iron, those established by the Food and Nutrition Board, Institute of Medicine, USA (23, 24) were used.

### Statistical Analysis

Descriptive statistics were used to present the number of participants and their percentage distribution by the status of additional source usage. Categorical variables by VFF and/or VMS status were compared using the Pearson Chi-square test; the results with *P* ≤ 0.05 were considered as statistically significant. The distributions of the amounts of vitamin and mineral intakes from an additional source were presented as a mean and standard deviation, as well as the 5th, 25th, 50th, 75th, and 95th percentiles (P). Statistical analyses were performed using the STATISTICA 13.0 PL software.

To compare the nutrient intakes in relation to DRI, the participants were classified into 7 categories (<20%, 20–39.9%, 40–59.9%, 60–79.9%, 80–99.9%, 100–119%, or >120% of DRI) based on vitamin and mineral intakes from VFFs and/or VMSs. Nutrients were classified into four levels of complementation from VFFs and/or VMSs:

level I—when the nutrient intake for more than 50% of participants did not exceed 20% of DRI;
level II—when 50% of participants consumed at least 20% of DRI;
level III—when 50% of participants consumed at least 40% of DRI;
level IV—when 50% of participants consumed at least 60% of DRI.

## Results

### Characteristics of VFF and/or VMS Use

The characteristics of the study population concerning VFF and/or VMS usage are presented in Table 1. The results indicate that the consumption of additional sources of vitamins and/or minerals was a common practice among adolescents. Only 12.3% of respondents used neither VFF nor VMS. Most often, the participants only used VFFs (70.0%); next, VFFs and VMSs were used simultaneously by 16.6% of respondents, while a few people used VMSs only. Age, socioeconomic status and the number of meals per day were factors that influenced the use of VFF and/or VMS. “None” users vs. “VFF only” users as well as “VFF and VMS” users were younger and more likely to consume at least 3 meals per day. Moreover, “none” users compared to “VFF only” users more often self-reported their socio-economic status as very good and good.

**Table 1:** Characteristics of the study population by socio-demographic factors, health status and dietary habits in relation to voluntarily fortified food (VFF) and/or vitamin/mineral supplements (VMS) usage.

**Parameter**	**Total *n* = 759**	**Usage (%)**				***P*-value^**b**^**
		**None *n* = 93**	**VFF only *n* = 532**	***VMS only^**a**^ n = 8***	**VFF and VMS *n* = 126**	
**SOCIO-DEMOGRAPHIC FACTORS**						
**Gender**						
Girls	66.8	65.6	66.0	*62.5*	71.4	0.487
Boys	33.2	34.4	34.0	*37.5*	28.6	
**Age category**						
13–16 y	30.8	9.7^A^	33.5^B^	*25.0*	35.7^B^	0.001
17–19 y	69.2	90.3	66.5	*75.0*	64.3	
**Residential area**						
rural	59.4	57.8	60.6	*87.5*	54.0	0.381
urban	40.6	42.2	39.4	*12.5*	46.0	
**Self-reported socioeconomic status**						
very good & good	61.4	69.3^A^	59.1^B^	*57.1*	65.8^A, B^	0.015
average	35.4	22.7	38.2	*42.9*	31.7	
poor	3.2	8.0	2.7	–	2.5	
**FACTORS RELATED TO HEALTH**						
**Self-reported physical activity level**						
low	7.1	11.6	6.4	–	7.3	0.160
moderate	69.1	58.2	58.4	*100*	62.1	
high	33.5	30.2	35.2	–	30.6	
**Body mass index**						
<18.5 kg/m2	12.8	12.5	14.6	–	6.3	0.162
18.5–24.9 kg/m2	73.1	71.6	72.1	*75.0*	78.6	
≥25 kg/m2	14.1	15.9	13.3	*25.0*	15.1	
**Self-reported health status**						
Very good & good	85.3	85.1	87.1	*50.0*	80.5	0.169
Average & poor	14.7	14.9	12.9	*50.0*	19.5	
**Self-reported chronic diseases**						
No	94.6	96.8	95.3	*87.5*	90.5	0.059
Yes	5.4	3.2	4.7	*12.5*	9.5	
**FACTORS RELATED TO DIETARY HABITS**						
**Number of meals/day**						
1–2	7.5	16.2^A^	6.8^B^	–	4.7^B^	0.019
3	32.9	24.7	34.9	*50.0*	29.4	
4	42.6	43.0	41.2	*37.5*	48.4	
≥5	17.0	16.1	17.1	*12.5*	17.5	
**Breakfast**						
Yes	68.8	61.3	69.6	*50.0*	72.0	0.404
No	15.9	17.2	15.8	*50.0*	13.6	
Irregular	15.3	21.5	14.6	–	14.4	
**Morning snack**						
Yes	46.5	46.1	47.4	*25.0*	44.5	0.734
No	25.0	29.2	24.0	*62.5*	23.5	
Irregular	28.5	24.7	28.6	*12.5*	32.0	
**Lunch**						0.193
Yes	89.4	87.1	88.8	*75.0*	94.4	
No	2.6	2.2	3.0	*25.0*	–	
Irregular	8.0	10.7	8.2	–	5.6	
**Afternoon snack**						
Yes	33.9	34.0	34.0	*42.9*	32.7	0.413
No	33.6	38.5	33.8	*42.9*	27.9	
Irregular	32.5	27.5	32.2	*14.2*	39.4	
**Dinner**						
Yes	73.4	65.2	74.0	*62.5*	77.6	0.076
No	10.8	14.1	11.3	*37.5*	4.8	
Irregular	15.8	20.7	14.7	–	17.6	

*n, number of people; VFF, voluntarily fortified food; VMS, vitamin/mineral supplement*.

a , group excluded from statistical analysis due to a small number of subjects;

b , test P-value was determined using the chi-square test;

A, B *different capital letters in superscript indicate statistically significant differences between subgroups; missing data: place of residential area (1.6%), socioeconomic status (10.3%), physical activity level (4.0%), body mass index (1.4%), health status (4.0%), consumption of breakfast (0.8%), morning snack (5.1%), lunch (0.7%), afternoon snack (9.9%), and dinner (1.4%). The italic values emphasize that the group (VFF only) excluded from statistical analysis due to a small number of subjects*.

**Figure 1:**
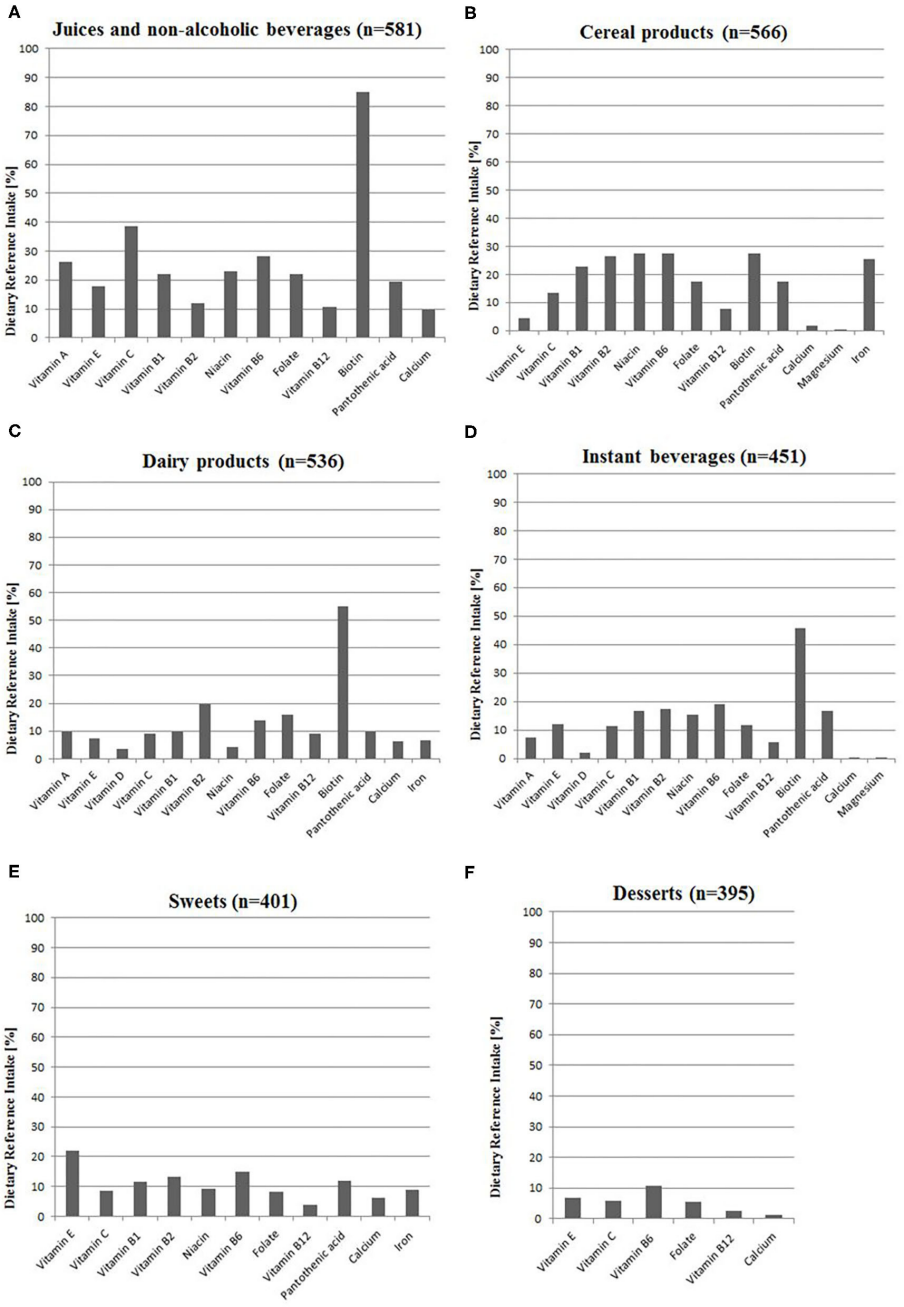
Intake of nutrients from voluntarily fortified foods as a percentage of Dietary Reference Intake (20) among VFF-consumers (*n* = 658): **(A)** Juices and non-alcoholic beverages, **(B)** Cereal products, **(C)** Dairy products, **(D)** Instant beverages, **(E)** Sweets, **(F)** Desserts. All calculations based on the median intake of nutrients from VFF groups; details are presented in Supplementary Table 1. n, number of people; VFF, voluntarily fortified food.

The most common groups of VFFs consumed by adolescents were juices and non-alcoholic beverages, cereal products as well as dairy products (88.3, 86.0, and 81.5%, respectively; Figure 1). Data on VFF consumption is partially overlapping with a group of adolescents (13–20 years old), which has been described in detail elsewhere (10).

We identified 103 different VMSs used by adolescents. Multivitamin and/or multimineral supplements were used most frequently, while single vitamin or single mineral were used less commonly. From specific nutrients, vitamin C was used most frequently (77.6% of VMS-users), while biotin was used least frequently (20.1%) (Supplementary Table 2). The reasons for MVS usage by the group of adolescents (13–20 years old) has been described in detail elsewhere (11).

### Comparison of Nutrient Intakes From VFFs and/or VMSs to Polish DRI

The participants consumed 12 vitamins and three minerals from VFFs that were added according to the producer's declarations. The respondents consumed the highest amounts of vitamins A, C, B_6_, B_12_, folate, pantothenic acid, and calcium (10–39% DRI) from juices and non-alcoholic beverages, as well as vitamins B_1_, B_2_, niacin, iron from cereal products (23–27% DRI). Sweets provided meaningful amounts of vitamin E (22% DRI). The consumption of fortified juices and non-alcoholic beverages, dairy products as well as instant beverages provided relatively high amounts of biotin in comparison to DRI (45–85%). Consumed fortified products hardly provided vitamin D and magnesium (Figure 1).

Among additional sources (VFF and/or VMS), almost all users (96–99% of them) consumed vitamin C, B_1_, B_2_, B_6_, B_12_, niacin, folic acid, and pantothenic acid from the mentioned products, while the least of users consumed magnesium (33%) and vitamin A (56%) (Table 2). The median intake of the majority of nutrients did not exceed DRI values, except biotin. The values of the 95th percentile of intake of the majority of vitamins (A, E, C, B_1_, B_2_, B_6_, B_12_, niacin, folic acid, biotin, pantothenic acid) and iron exceeded DRI values. More details on the distribution of the nutrient intake from the specific VFFs as well as from VMSs are presented in Supplementary Tables 1, 2.

**Table 2:** Distribution of nutrient intakes from additional sources of vitamins and/or minerals in adolescents (*n* = 666).

**Nutrient**	***n* (%)^**a**^**	**Mean ± SD^**b**^**	**Intake percentiles**^****b****^					**Dietary Reference Intake (DRI)**	**Tolerable upper intake levels**
			**5**	**25**	**50**	**75**	**95**	**EAR^**c**^ or AI^**d**^**	**UL^**e**^**
Vitamin A (μg RE /d) ^f^	372 (55.9)	202 ± 327	12.0	46.9	104	226	800	490–630 ^c^	2,000–3,000
Vitamin E (mg/d)	584 (87.7)	4.4 ± 4.8	0.4	1.3	3.0	6.0	13.3	8–10 ^d^	220–300
Vitamin D (μg/d)	488 (73.3)	1.3 ± 2.6	0.1	0.3	0.6	1.3	5.0	15 ^d^	100
Vitamin C (mg/d)	649 (97.4)	67.6 ± 71.0	6.6	22.3	47.7	91.2	185	55–75 ^c^	1,200–2,000
Vitamin B_1_ (mg/d)	650 (97.6)	0.8 ± 0.7	0.1	0.3	0.6	1.0	1.8	0.9–1.1 ^c^	n/e
Vitamin B_2_ (mg/d)	653 (98.0)	0.9 ± 0.80	0.1	0.4	0.7	1.3	2.3	0.9–1.1 ^c^	n/e
Niacin (mg/d)	645 (96.8)	7.8 ± 6.4	0.7	3.4	6.1	10.9	18.9	11–12 ^c^	20–35
Vitamin B_6_ (mg/d)	652 (97.9)	1.5 ± 7.9	0.2	0.5	0.9	1.8	2.8	1.0-1.1 ^c^	15–25
Folic acid (μg/d)	651 (97.7)	148 ± 118	15.1	62.6	121	215	345	n/e	600–1,000
Folate (μg DFE/d) ^g^		251 ± 201	25.7	106	206	365	587	320–330^c^	n/e
Vitamin B_12_ (μg/d)	656 (98.5)	0.9 ± 0.7	0.1	0.3	0.7	1.2	2.1	2 ^c^	n/e
Biotin (μg/d)	511 (76.7)	35.8 ± 28.9	3.4	12.3	30.4	49.9	96.3	25–30 ^d^	n/e
Pantothenic acid (mg/d)	642 (96.4)	3.4 ± 3.1	0.4	1.3	2.5	4.7	8.6	5 ^d^	n/e
Calcium (mg/d)	618 (92.8)	211 ±184	11.1	77.1	159	300	573	800–1,100 ^c^	2,500–3,000
Magnesium (mg/d)	220 (33.0)	18.0 ± 47.3	0.3	0.9	2.1	4.5	100	255–340 ^c^	350
Iron (mg/d)	564 (84.7)	3.2 ± 3.4	0.2	1.1	2.3	3.8	9.4	6–8 ^c^	40–45

*AI, Adequate Intake; DFE, dietary folate equivalents; EAR, Estimated Average Requirement; n, number of people; n/e, not established; RE, retinol equivalents; SD, standard deviation; UL, Tolerable Upper Intake Level; VFF, voluntarily fortified food; VMS, vitamin/mineral supplement*.

a calculated in relation to all VFF and/or VMS users (n = 666);

b calculated in relation to those who intake a specific nutrient from VFFs and/or VMSs;

c EAR/^d^ AI—given as a range of values depending on the age and sex, according to the Polish DRI (20);

e UL values established for vitamins A, E, D, B6, and folic acid (21, 22), and for vitamin C, niacin, calcium, magnesium and iron (23, 24), UL for vitamin A applies only to retinol forms;

f RE expressed as 1 μg RE equals 1 μg of retinol, 6 μg of β-carotene, and 12 μg of other provitamin A carotenoids (20);

g DFE was calculated by multiplying the folic acid amount by a factor of 1.7 (taking into account the differences in bioavailability) (23).

c, d *represent different levels of the Dietary Reference Intake*.

The analysis of the distribution of nutrient intakes from additional sources (VFF and/or VMS) by percentages of DRI is presented in Table 3. Adolescents consumed nutrients from additional sources at different levels: level I—vitamins A, D, calcium and magnesium (>50% of adolescents did not exceed 20% of DRI); level II—vitamins E, B_12_, and iron (>50% of respondents consumed at least 20% of DRI); level III—niacin and pantothenic acid (>50% of respondents consumed at least 40% of DRI); level IV—vitamins C, B_1_, B_2_, B_6_, folate, biotin (>50% of participants consumed at least 60% of DRI).

**Table 3:** Distribution of adolescents (*n* = 666) by nutrient intakes from additional sources of vitamins and/or minerals by Dietary Reference Intake (DRI) (%).

**Nutrient**	**Distribution of adolescents (*****n*** **=** **666) meeting DRI (%)** ^****a****^							**Percentage of adolescents > UL^**b**^**
	**<20% DRI**	**20–39.9% DRI**	**40–59.9% DRI**	**60–79.9% DRI**	**80–99.9% DRI**	**100–119.9% DRI**	**≥120% DRI**	
Vitamin A	52.7	21.2	9.1	3.8	2.7	1.6	8.9	0.2
Vitamin E	31.9	24.1	13.5	9.9	7.4	3.3	9.9	0
Vitamin D	92.2	4.9	0.6	1.7	0.2	0	0.4	0
Vitamin C	10.9	15.7	13.7	9.9	7.6	8.0	34.2	0
Vitamin B_1_	12.9	18.0	17.2	13.9	9.5	6.2	22.3	n/a
Vitamin B_2_	11.8	15.2	12.5	12.5	12.7	7.4	27.9	n/a
Niacin	15.5	20.2	20.0	11.9	9.9	6.2	16.3	1.1
Vitamin B_6_	9.1	12.4	11.8	12.4	9.7	5.8	38.8	0.2
Folate ^c^	15.1	17.8	13.5	14.6	8.8	9.7	20.5	0.2
Vitamin B_12_	30.4	26.2	18.9	11.9	6.1	3.8	2.7	n/a
Biotin	9.2	11.9	9.6	8.4	6.5	5.7	48.7	n/a
Pantothenic acid	18.4	23.2	17.5	10.3	7.6	5.9	17.1	n/a
Calcium	59.0	26.9	10.2	2.8	0.6	0.3	0.2	0
Magnesium	89.1	6.8	2.3	0.5	0.9	0	0.4	0
Iron	36.9	27.3	18.1	7.6	3.4	2.3	4.4	0

*AI, Adequate Intake; DFE, dietary folate equivalents; EAR, Estimated Average Requirement; n, number of people; n/a, not applicable; UL, Tolerable Upper Intake Level; VFF, voluntarily fortified food; VMS, vitamin/mineral supplement*.

a EAR/ AI—given as a range of values depending on the age and sex, according to the Polish DRI (20);

b UL values established for vitamins A, E, D, B6, and folic acid (21, 22), and for vitamin C, niacin, calcium, magnesium and iron (23, 24), UL for vitamin A applies only to retinol forms; percentage of adolescents > UL value calculated in relation to all VFF and/or VMS users (n = 666);

c *given as DFE to compare with DRI, folic acid was used to compare with UL (23)*.

**Figure 2:**
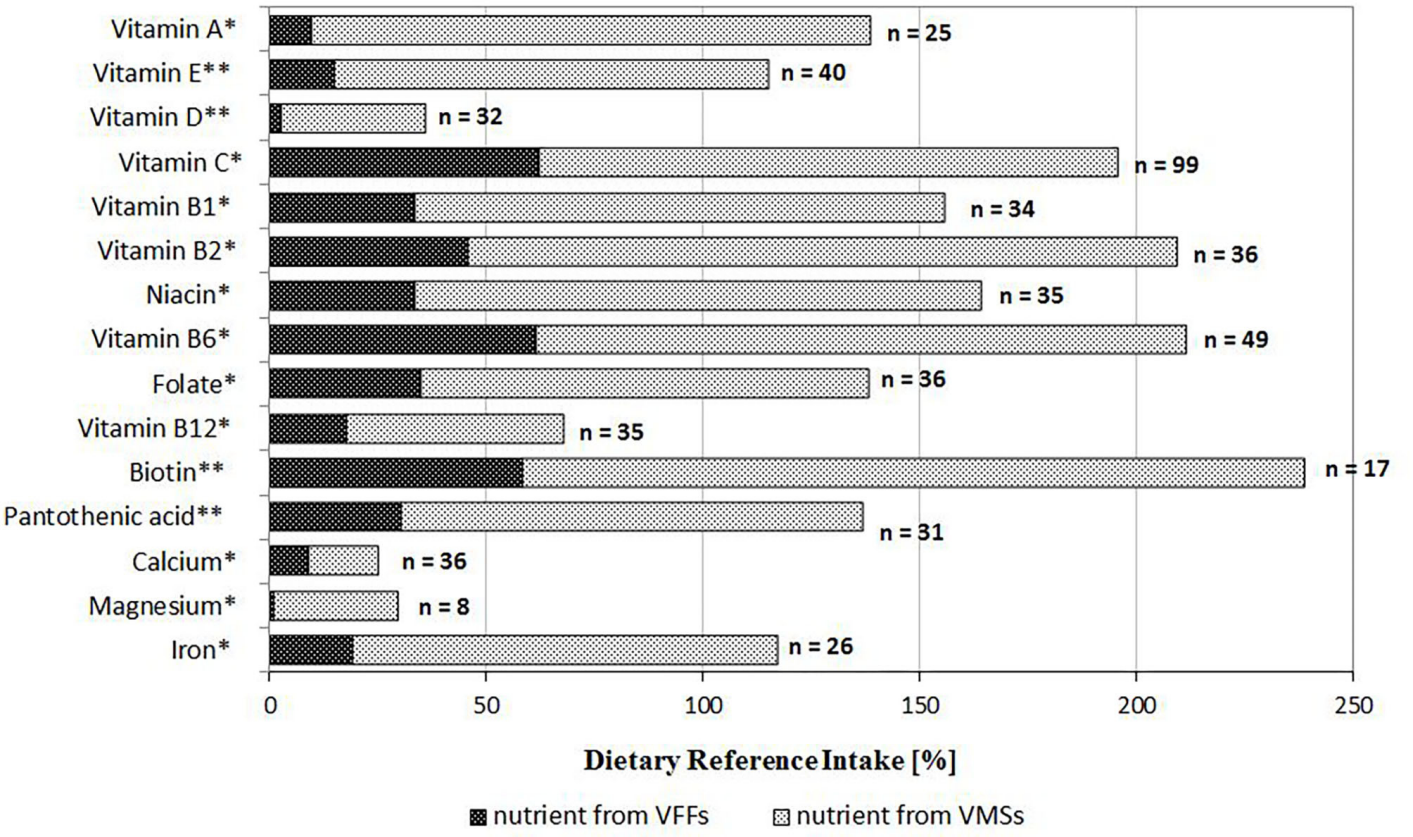
Intake of nutrients from additional sources as a percentage of Dietary Reference Intake among adolescents who used both products (VFF and VMS) simultaneously (*n* = 126); *EAR or **AI according to the Polish DRI (20). AI, Adequate Intake; EAR, Estimated Average Requirement; VFF, voluntarily fortified food; VMS, vitamin/mineral supplement.

In a subgroup of respondents who used VFFs and VMSs simultaneously (*n* = 126), some nutrients (i.e., vitamins C, B_1_, B_2_, B_6_, niacin and biotin) were consumed in amounts ≥150% of DRI (Figure 2). Compared to VFFs, VMS consumption provided more than five times higher amounts of vitamin A, E, D, magnesium and iron. Overall, the average consumption of the majority of nutrients from VFFs did not exceed 40% of DRI, except biotin, vitamin B_2_, B_6_, and C.

The ULs were exceeded in single cases. It was observed that with VFFs and/or VMSs, seven respondents (1.1% of users) consumed niacin, and some individuals (0.2%) consumed vitamin A, B_6_, and folic acid in amounts exceeding the ULs (Table 3). The highest intake of these vitamins was derived mostly from VMS. For example, 19-years old women exceeded the ULs for niacin (intake 60 mg/day), vitamin A (4,032 μg/day), and folic acid (1,200 μg/day) via using two tablets of a multivitamin supplement three times per day, although the manufacturer's recommendation was using one pill per day.

## Discussion

In our study, the quantitative consumption of nutrients from additional sources (VFF and/or VMS) by adolescents was analyzed. It was found that more than 86% of all participants consumed VFFs and more than 17% used VMSs. Frequent consumption of VFF is a common behavior among young people in some countries, such as Germany (25), Ireland (26), and the United States (27), but not in Japan (28). Nevertheless, consumers are often unaware that they consume fortified products (10). The prevalence of VMS use by adolescents differs between countries. Depending on various factors, such as age/sex groups, socioeconomic status and physical activity level, the period of research and product availability on the market ranges from 6 to 45% in Germany (25), the United States (27), Finland (29), and Slovenia (30).

The results of our study showed that adolescents consumed nutrients from additional sources at various levels, at least 50% of adolescents consumed vitamin C and the majority of B vitamins from VFFs and/or VMSs in amounts, which may prevent inadequate intake, i.e., above 40% of DRI (level III and IV). On the other hand, the provided amounts of vitamin A, E, B_12_, calcium, and iron were small (>50% of respondents did not exceed 40% of DRI). In the case of vitamin D and magnesium, the amounts from VFFs and/or VMSs were negligible (>85% of adolescents did not exceed 20% of DRI), and it would not prevent an inadequate intake (level I and II). Similarly, in our previous survey conducted among school children (*n* = 677, 6–12 years), the provided amounts of vitamin D and calcium from VFF were very small (11). Although in Poland, fat spreads are mandatorily fortified with vitamins A and D, these products are rarely consumed by adolescents; <1/6 of them declared frequent usage of margarines (31). Inadequate dietary intake of certain nutrients, such as vitamin D, calcium and magnesium during puberty may result in insufficient bone mineralization and increases the risk of developing osteoporosis. In the study conducted in Eastern Poland among 10–15-year old girls (*n* = 461), large deficiencies of vitamin D and calcium in diets (intakes at an average level of 40% DRI for both nutrients) were observed (32). Therefore, it seems that the intake of nutrients from additional sources associated with bone mineralization should be promoted via VFFs and/or VMSs. According to the Practical Guidelines for healthcare professionals in Central Europe, dietary supplementation has been recommended as an essential strategy to achieve an optimal vitamin D status in the population, including adolescents (33). While in our study, only 6% of VMSs used by adolescents contained vitamin D, and 63% of respondents consumed VFF with vitamin D. In the National Teens Food Survey 2005–2006 conducted among Irish teenagers (*n* = 441; 13–17 years), 15% of respondents declared using vitamin D supplements and more than 60% consumed foods fortified with vitamin D. Moreover, fortified foods had a relatively important contribution (13–25%) to the total intakes of many micronutrients in Irish teenagers, particularly iron, B vitamins and vitamin D, but a low contribution for calcium (7%) (26, 34). Older data from the DONALD study of German children and adolescents (*n* = 931, 2–18 years) conducted in 1986–2003 showed that fortified foods had a limited impact on raising the total vitamin D intake, while supplements considerably helped increase vitamin D intake (25). A study on the intake of vitamin A, D, E, and K (VITADEK study) analyzed the total intake of fat-soluble vitamins (i.e., regular diet, fortified food, dietary supplements) in the Belgian population aged 3–64 (*n* = 3,200) in 2014. In a group of 11–17-year-old adolescents, the contribution of VFFs and/or VMSs to the total vitamin A and E intakes was low; however, contribution to the total vitamin D intake was more considerable, i.e., up to 12% for VFFs and 41% for VMSs. Overall, the fortification and supplementation practices were inadequate to eradicate suboptimal intakes of vitamins A and D in the Belgian population (4). In the Japanese National Health and Nutrition Survey (*n* = 64,624; aged ≥ 1 year) conducted in 2003–2009 (28), the use of VFFs and/or VMSs had a minor effect on the median and the 95th percentile of the total vitamin E intake in the population.

In a study derived from the national representative survey in nine European countries (35), patterns of voluntary fortification and food supplements vary widely between countries, and VMSs were responsible for the largest differences in total nutrient intakes. Fortified foods did not significantly contribute to higher intakes of nutrients. Firstly, this may be explained by the fairly moderate levels of nutrients in fortified products, and secondly, by relatively low intakes of VFFs even in high consumers of those foods. In the National Health and Nutrition Examination Survey 2003–2006 (27) in US children and adolescents (*n* = 7,250, aged 2–18 years), the consumption of fortified food was influenced more by intakes of vitamins than minerals, but even with the increased intakes of vitamins A, C, and D from fortified foods, substantial percentages of most age/sex subgroups had intakes of these vitamins from the diet below the DRI. In addition, fortification had a minimal impact on the usual intake for several shortfall nutrients, such as calcium, magnesium and vitamin E (36).

High VFF and VMS consumption over a prolonged period can potentially lead to unacceptably high intakes of micronutrients that exceed the UL levels and may be associated with adverse health effects (21). Our results indicated that the risk of an excessive intake of vitamins and minerals through VFFs and/or VMSs was relatively low for the majority of nutrients. However, we observed intakes of niacin, vitamin A, vitamin B_6_ and folic acid above the UL values up to 1.1% of the study group. In a subgroup of respondents who used both VFFs and VMSs simultaneously (16.6% of all respondents), a higher risk of overconsumption of nutrients was associated with using VMSs than with VFF consumption. In general, total nutrient intakes (including VFFs and/or VMSs) in European consumers do not exceed the UL; however, for some nutrients, i.e., retinol (3, 4, 25, 35), folic acid (3, 25), vitamin B_6_ (3), vitamin D, vitamin E, vitamin C (25), iodine (3, 35), zinc, magnesium, copper (35), the UL is exceeded in individual cases; the most pronounced subgroup in the populations was young children (25, 35). In the US study among children and adolescents, there was no concern about fortification contributing to intakes above the UL for most micronutrients, except folic acid, niacin, and zinc in the youngest examined subgroup (27); although, among supplement users aged 14–18, the prevalence of total intakes above the UL for iron, zinc and folic acid increased up to 13% (36). Patterns of fortified food and dietary supplement consumption might evolve and require constant monitoring since, in many European countries, information on the consumption of these products is not available in the dietary survey (1, 35).

The novelty of this study was a new approach to the classification of nutrient intake from additional sources in the context of potential deficiency complementation. The introduction of the four levels of classification allowed us to indicate nutrients, which were supplemented only at a negligible level as well as those of which supplementation seemed to be sufficient. The results might be helpful in determining improvements to public health nutrition policy.

Major strengths of this study include the specific design to collect accurate data on the VFF and/or VMS consumption, a specially developed FFQ with an open list of fortified foodstuffs, products that were present on the market at the time of the dietary survey; every VMS used by the respondent was identified on the market as well. Due to the fact that VFF and VMS markets have been changing dynamically, collecting accurate and updated data about these products poses a challenge for researchers. Therefore, these products are often overlooked or underestimated, and as a consequence, the number of respondents with micronutrient deficiencies identified in dietary studies can be overestimated.

The limitation of this study was that the population under study might not be representative of all school adolescents from Central-Eastern Poland because the study was carried out only in those schools in which the headteacher gave consent to it and not all respondents returned the completed questionnaires. An overrepresentation of girls compared to boys, as well as more respondents living in rural areas compared to urban areas, were observed in the study; both characteristics were not consistent with the distribution of adolescents of this age in Poland (12). It is also possible that participants with a more health-oriented lifestyle were more likely to participate in the study. Moreover, the market with added nutrients is constantly developing; therefore, there was a possibility of omitting some products in the FFQ. Although the participants had the possibility to add extra products available on the market to the FFQ list, they were often unaware which products were fortified and therefore they did not indicate them. Furthermore, in the study, the mandatory fortified products available in Poland, i.e., iodised salt and margarine (fat spreads) fortified with vitamin A and vitamin D, were not taken into account. In addition, this survey has focused only on VFFs and/or VMs consumption, which has not allowed to estimate the contribution of the nutrient intakes from these products in the total intake.

## Conclusions

The results of the study show that the consumption of VFFs was a common behavior in adolescents, but VMSs were used only by less than one-fifth of them. At least 50% of adolescents consumed amounts of vitamin C and the majority of B vitamins from VFFs and/or VMSs that may prevent an inadequate intake of these nutrients. The risk of excessive intake of micronutrients with VFFs and/or VMSs was relatively low; while a higher risk of overconsumption was associated with using VMSs than VFFs. As these products' market continues to expand, more careful monitoring of these practices is needed in the population.

## Data Availability Statement

The datasets generated for this study are available on request to the corresponding author.

## Ethics Statement

The study was reviewed and approved by the Ethical Committee of the Warsaw University of Life Sciences (Resolution No. 103 09_1/2016). Parents were informed on involving children in the conducted survey. By completing the questionnaires, the respondents agreed to participate in the study.

## Author Contributions

ES, BP, and JK was responsible for the manuscript concept and performed statistical analysis. BP and JK were responsible for data collection and were responsible for critical revision of the manuscript for important intellectual content. ES, OJ, SJ, KK-B, and KR for preparing the database. ES drafted and wrote the manuscript. All authors approved the final version of this manuscript.

## Conflict of Interest

The authors declare that the research was conducted in the absence of any commercial or financial relationships that could be construed as a potential conflict of interest.
