# Supplementary material for: Intake of Vitamins and Minerals From Voluntarily Fortified Foods and/or Dietary Supplements in School Adolescents in Central-Eastern Poland
Source: Front Public Health. 2020 Oct 9;8:504015. doi: 10.3389/fpubh.2020.504015 (PMC7581890; doi:10.3389/fpubh.2020.504015)
Supplement: Supplementary file 1 [file Table_1.DOCX]

**Supplementary Materials** Table S1. Distribution of nutrient intakes from voluntarily fortified foods (VFFs) by adolescents (n=658)

| Nutrient and food groups | n=658 | Mean ± SD | Intake percentiles | | | | |
| --- | --- | --- | --- | --- | --- | --- | --- |
|  |  |  | 5 | 25 | 50 | 75 | 95 |
| **Vitamin A (µg/d)** | **348** | **113 ± 101** | **11.1** | **40.0** | **80.0** | **160** | **320** |
| Juices and non-alcoholic beverages | 115 | 159 ±103 | 34.3 | 85.7 | 129 | 226 | 396 |
| Dairy products | 246 | 66.8 ± 50.0 | 8.00 | 32.0 | 51.5 | 96.0 | 140 |
| Instant beverages | 87 | 54.2 ± 51.1 | 5.71 | 22.9 | 45.7 | 68.6 | 160 |
| **Vitamin E (mg/d)** | **574** | **3.66 ± 3.42** | **0.34** | **1.07** | **2.65** | **5.25** | **10.7** |
| Cereal products | 27 | 0.66 ± 0.63 | 0.09 | 0.18 | 0.36 | 0.73 | 1.82 |
| Juices and non-alcoholic beverages | 237 | 1.78 ± 1.24 | 0.25 | 0.71 | 1.56 | 2.14 | 4.28 |
| Dairy products | 248 | 0.80 ± 0.62 | 0.09 | 0.37 | 0.70 | 1.13 | 1.70 |
| Sweets | 203 | 3.01 ± 3.33 | 0.11 | 0.44 | 1.77 | 4.70 | 9.00 |
| Instant beverages | 413 | 1.62 ± 1.54 | 0.14 | 0.41 | 1.04 | 2.39 | 4.71 |
| Desserts | 211 | 0.89 ± 0.82 | 0.13 | 0.27 | 0.54 | 1.07 | 2.68 |
| **Vitamin D (µg/d)** | **475** | **0.72 ± 0.69** | **0.06** | **0.23** | **0.54** | **0.95** | **2.07** |
| Dairy products | 451 | 0.69 ± 0.65 | 0.06 | 0.21 | 0.53 | 0.93 | 1.84 |
| Instant beverages | 87 | 0.34 ± 0.32 | 0.04 | 0.14 | 0.29 | 0.43 | 1.00 |
| **Vitamin C (mg/d)** | **644** | **49.8 ± 42.0** | **5.01** | **18.9** | **37.6** | **71.6** | **124** |
| Cereal products | 502 | 9.46 ± 7.72 | 0.91 | 4.37 | 7.84 | 13.3 | 21.8 |
| Juices and non-alcoholic beverages | 508 | 34.1 ± 35.7 | 3.54 | 10.7 | 21.4 | 42.9 | 99.0 |
| Dairy products | 307 | 6.49 ± 5.12 | 0.71 | 2.86 | 5.71 | 9.00 | 17.4 |
| Sweets | 204 | 15.3 ± 19.8 | 0.26 | 1.66 | 4.76 | 25.5 | 53.1 |
| Instant beverages | 355 | 10.0 ± 9.04 | 1.05 | 2.63 | 6.86 | 15.0 | 28.6 |
| Desserts | 213 | 5.77 ± 7.40 | 0.81 | 1.63 | 3.25 | 6.50 | 16.3 |
| **Vitamin B_1_ (mg/d)** | **646** | **0.68 ± 0.53** | **0.07** | **0.29** | **0.53** | **0.94** | **1.69** |
| Cereal products | 566 | 0.25 ± 0.23 | 0.02 | 0.10 | 0.21 | 0.36 | 0.68 |
| Juices and non-alcoholic beverages | 236 | 0.25 ± 0.17 | 0.04 | 0.10 | 0.20 | 0.30 | 0.60 |
| Dairy products | 302 | 0.11 ± 0.08 | 0.02 | 0.05 | 0.09 | 0.16 | 0.29 |
| Sweets | 349 | 0.27 ± 0.39 | 0.01 | 0.05 | 0.11 | 0.26 | 1.04 |
| Instant beverages | 450 | 0.23 ± 0.22 | 0.02 | 0.06 | 0.15 | 0.33 | 0.71 |
| **Vitamin B_2_ (mg/d)** | **650** | **0.81 ± 0.60** | **0.08** | **0.33** | **0.66** | **1.09** | **2.05** |
| Cereal products | 566 | 0.31 ± 0.27 | 0.02 | 0.12 | 0.25 | 0.42 | 0.80 |
| Juices and non-alcoholic beverages | 53 | 0.14 ± 0.10 | 0.02 | 0.07 | 0.11 | 0.20 | 0.33 |
| Dairy products | 487 | 0.25 ± 0.23 | 0.03 | 0.09 | 0.19 | 0.34 | 0.61 |
| Sweets | 349 | 0.33 ± 0.48 | 0.01 | 0.05 | 0.12 | 0.30 | 1.27 |
| Instant beverages | 450 | 0.26 ± 0.24 | 0.03 | 0.08 | 0.17 | 0.38 | 0.74 |
| **Niacin (mg/d)** | **641** | **6.80 ± 4.87** | **0.73** | **3.18** | **5.54** | **9.64** | **16.7** |
| Cereal products | 566 | 3.18 ± 2.74 | 0.21 | 1.31 | 2.62 | 4.48 | 8.07 |
| Juices and non-alcoholic beverages | 239 | 3.13 ± 2.25 | 0.39 | 1.27 | 2.55 | 3.86 | 7.71 |
| Dairy products | 248 | 0.52 ± 0.45 | 0.07 | 0.23 | 0.46 | 0.69 | 1.14 |
| Sweets | 341 | 1.56 ± 1.58 | 0.11 | 0.45 | 1.00 | 2.22 | 4.17 |
| Instant beverages | 413 | 2.81 ± 2.71 | 0.27 | 0.69 | 1.68 | 4.20 | 8.57 |
| **Vitamin B_6_ (mg/d)** | **648** | **1.04 ± 0.76** | **0.14** | **0.44** | **0.83** | **1.50** | **2.53** |
| Cereal products | 566 | 0.35 ± 0.30 | 0.02 | 0.14 | 0.29 | 0.50 | 0.90 |
| Juices and non-alcoholic beverages | 290 | 0.37 ± 0.31 | 0.04 | 0.14 | 0.28 | 0.54 | 0.99 |
| Dairy products | 303 | 0.19 ± 0.15 | 0.02 | 0.09 | 0.14 | 0.27 | 0.46 |
| Sweets | 388 | 0.34 ± 0.43 | 0.01 | 0.06 | 0.15 | 0.39 | 1.21 |
| Instant beverages | 450 | 0.32 ± 0.31 | 0.03 | 0.09 | 0.20 | 0.47 | 1.00 |
| Desserts | 212 | 0.17 ± 0.16 | 0.03 | 0.05 | 0.11 | 0.21 | 0.54 |
| **Folic acid (µg /d)** | **647** | **133 ± 96.5** | **14.6** | **58.0** | **115** | **194** | **323** |
| Cereal products | 566 | 40.1 ± 35.7 | 3.34 | 15.2 | 33.6 | 51.0 | 103 |
| Juices and non-alcoholic beverages | 291 | 45.4 ± 35.9 | 4.29 | 19.3 | 41.6 | 64.0 | 107 |
| Dairy products | 362 | 42.7 ± 38.7 | 2.68 | 14.3 | 30.4 | 64.0 | 117 |
| Sweets | 388 | 40.3 ± 54.9 | 1.43 | 7.50 | 15.9 | 46.6 | 154 |
| Instant beverages | 413 | 36.4 ± 33.5 | 3.81 | 11.0 | 22.9 | 53.3 | 101 |
| Desserts | 212 | 18.0 ± 16.5 | 2.71 | 5.41 | 10.8 | 21.6 | 54.1 |
| **Vitamin B_12_ (µg/d)** | **652** | **0.77 ± 0.58** | **0.08** | **0.29** | **0.63** | **1.11** | **1.94** |
| Cereal products | 565 | 0.19 ± 0.16 | 0.01 | 0.07 | 0.15 | 0.25 | 0.51 |
| Juices and non-alcoholic beverages | 292 | 0.24 ± 0.20 | 0.02 | 0.11 | 0.24 | 0.32 | 0.62 |
| Dairy products | 488 | 0.27 ± 0.26 | 0.03 | 0.08 | 0.18 | 0.40 | 0.76 |
| Sweets | 371 | 0.22 ± 0.31 | 0.01 | 0.04 | 0.08 | 0.26 | 0.84 |
| Instant beverages | 413 | 0.22 ± 0.27 | 0.02 | 0.05 | 0.11 | 0.27 | 0.83 |
| Desserts | 212 | 0.09 ± 0.08 | 0.01 | 0.03 | 0.05 | 0.11 | 0.27 |
| **Biotin (µg/d)** | **501** | **32.9 ± 26.1** | **3.43** | **11.4** | **28.6** | **44.1** | **88.7** |
| Cereal products | 18 | 7.96 ± 6.54 | 1.29 | 1.83 | 7.71 | 10.3 | 25.7 |
| Juices and non-alcoholic beverages | 232 | 26.6 ± 18.7 | 4.01 | 10.6 | 21.5 | 32.1 | 64.3 |
| Dairy products | 246 | 18.2 ± 14.0 | 1.61 | 9.14 | 13.7 | 25.1 | 40.0 |
| Sweets ^1^ | 5 | 3.78 ± 4.67 | - | - | 1.61 | - | - |
| Instant beverages | 324 | 17.6 ± 15.8 | 1.78 | 5.53 | 11.6 | 24.5 | 49.9 |
| **Pantothenic acid (mg/d)** | **636** | **3.05 ± 2.55** | **0.33** | **1.20** | **2.27** | **4.29** | **8.02** |
| Cereal products | 522 | 1.00 ± 0.82 | 0.09 | 0.43 | 0.87 | 1.47 | 2.40 |
| Juices and non-alcoholic beverages | 233 | 1.10 ± 0.74 | 0.16 | 0.52 | 0.96 | 1.29 | 2.57 |
| Dairy products | 301 | 0.67 ± 0.52 | 0.08 | 0.31 | 0.49 | 0.97 | 1.58 |
| Sweets | 388 | 1.46 ± 2.04 | 0.06 | 0.27 | 0.59 | 1.76 | 5.45 |
| Instant beverages | 355 | 1.11 ± 0.96 | 0.11 | 0.29 | 0.84 | 1.71 | 2.98 |
| **Calcium (mg/d)** | **613** | **199 ± 178** | **10.9** | **68.9** | **147** | **293** | **531** |
| Cereal products | 396 | 33.8 ± 43.9 | 0.02 | 8.40 | 17.5 | 42.9 | 119 |
| Juices and non-alcoholic beverages | 311 | 143 ± 136 | 17.1 | 42.9 | 103 | 180 | 396 |
| Dairy products | 348 | 84.8 ± 94.6 | 9.00 | 26.5 | 63.0 | 108 | 248 |
| Sweets | 317 | 86.7 ± 75.7 | 8.57 | 34.3 | 68.6 | 116 | 240 |
| Instant beverages | 109 | 4.34 ± 3.30 | 0.63 | 1.50 | 3.75 | 6.85 | 10.0 |
| Desserts | 303 | 21.3 ± 24.4 | 2.87 | 8.63 | 11.5 | 23.0 | 57.5 |
| **Magnesium (mg/d)** | **186** | **2.06 ± 1.71** | **0.27** | **0.75** | **1.80** | **3.00** | **5.43** |
| Cereal products | 89 | 1.92 ± 1.61 | 0.15 | 0.90 | 1.50 | 2.70 | 6.00 |
| Dairy products ^1^ | 8 | 1.13 ± 1.49 | - | - | 0.46 | - | - |
| Instant beverages | 107 | 1.82 ± 1.39 | 0.27 | 0.64 | 1.29 | 2.14 | 4.28 |
| **Iron (mg/d)** | **557** | **2.58 ± 2.09** | **0.21** | **1.02** | **2.09** | **3.57** | **6.65** |
| Cereal products | 498 | 2.25 ± 1.85 | 0.21 | 1.02 | 1.98 | 3.06 | 5.28 |
| Juices and non-alcoholic beverages ^1^ | 5 | 0.46 ± 0.70 | - | - | 0.20 | - | - |
| Dairy products | 39 | 0.68 ± 0.72 | 0.13 | 0.19 | 0.48 | 0.69 | 2.68 |
| Sweets | 293 | 1.00 ± 0.97 | 0.09 | 0.35 | 0.70 | 1.40 | 2.45 |

^1^ the distribution is not presented due to a small number of consumers

Table S2. Distribution of nutrient intakes from vitamin/mineral supplements (VMSs) by adolescents (n=134)

| Nutrient | n=134 | Mean ± SD | Intake percentiles | | | | |
| --- | --- | --- | --- | --- | --- | --- | --- |
|  |  |  | 5 | 25 | 50 | 75 | 95 |
| Vitamin A (µg RE/d) ^1^ | 50 | 717 ± 609 | 105 | 400 | 708 | 800 | 1600 |
| Vitamin E (mg/d) | 50 | 9.38 ± 9.41 | 0.25 | 5.00 | 9.50 | 10.0 | 20.0 |
| Vitamin D (µg/d) | 45 | 6.06 ± 6.07 | 0.08 | 2.50 | 5.00 | 7.50 | 10.8 |
| Vitamin C (mg/d) | 104 | 114 ± 109 | 30.0 | 60.0 | 86.7 | 111 | 360 |
| Vitamin B_1_ (mg/d) | 38 | 1.47 ± 1.30 | 0.50 | 1.00 | 1.18 | 1.40 | 3.30 |
| Vitamin B_2_ (mg/d) | 39 | 1.85 ± 1.27 | 0.50 | 1.28 | 1.60 | 2.00 | 6.00 |
| Niacin (mg/d) | 39 | 16.4 ± 10.9 | 5.00 | 10.0 | 14.3 | 18.0 | 48.0 |
| Vitamin B_6_ (mg/d) | 53 | 5.63 ± 27.3 | 0.13 | 1.05 | 1.70 | 2.00 | 5.00 |
| Folic acid (µg/d) | 40 | 261 ± 198 | 100 | 200 | 200 | 200 | 600 |
| Vitamin B_12_ (µg/d) | 39 | 1.41 ± 1.00 | 0.50 | 1.00 | 1.00 | 1.50 | 3.00 |
| Biotin (µg/d) | 27 | 67.4 ± 39.7 | 16.0 | 37.5 | 50.0 | 100 | 150 |
| Pantothenic acid (mg/d) | 37 | 5.93 ± 6.10 | 1.0 | 3.00 | 5.93 | 6.00 | 18.0 |
| Calcium (mg/d) | 41 | 197 ±171 | 59.0 | 108 | 177 | 209 | 560 |
| Magnesium (mg/d) | 42 | 84.8 ± 78.7 | 0.12 | 41.7 | 68.3 | 100 | 270 |
| Iron (mg/d) | 33 | 10.1 ± 5.68 | 4.00 | 6.15 | 7.83 | 12.7 | 24.0 |

^1^ RE (retinol equivalents) express as 1 μg RE equals 1 μg  of  retinol,  6 μg of  β-carotene and 12 μg of other provitamin A carotenoids
